# Identification and Validation of Reference Genes for qPCR Detection of Serum microRNAs in Colorectal Adenocarcinoma Patients

**DOI:** 10.1371/journal.pone.0083025

**Published:** 2013-12-11

**Authors:** Guixi Zheng, Haiyan Wang, Xin Zhang, Yongmei Yang, Lili Wang, Lutao Du, Wei Li, Juan Li, Ailin Qu, Yimin Liu, Chuanxin Wang

**Affiliations:** Department of Clinical Laboratory, Qilu Hospital of Shandong University, Jinan, Shandong Province, China; University of Barcelona, Spain

## Abstract

Serum microRNAs (miRNAs) have become a highlighted research hotspot, especially for their great potential as a novel promising non-invasive biomarker in cancer diagnosis. The most frequently used approach for serum miRNAs detection is quantitative real time polymerase chain reaction (qPCR). In order to obtain reliable qPCR data of miRNAs expression, the use of reference genes as endogenous control is undoubtly necessary. However, no systematic evaluation and validation of reference genes for normalizing qPCR analysis of serum miRNAs has been reported in colorectal adenocarcinoma. We firstly profiled pooled serum of colorectal adenocarcinoma, colorectal adenoma and healthy controls and selected a list of 13 miRNAs as candidate reference genes. U6 snRNA (U6) and above-mentioned 13 miRNAs were included in further confirmation by qPCR. As a result, 5 miRNAs (miR-151a-3p, miR-4446-3p, miR-221-3p, miR-93-5p and miR-3184-3p) were not detected in all samples and 2 miRNAs (miR-197-3p and miR-26a-5p) were relatively low with median Cq more than 35, and were excluded from further stability analysis. Then variable stability of other 6 miRNAs (miR-103b, miR-484, miR-16-5p, miR-3615, miR-18a-3p and miR-191-5p) and U6 were evaluated using two algorithms: geNorm and NormFinder which both identified miR-191-5p as the most stably expressed reference gene and selected miR-191-5p and U6 as the most stable pair of reference genes. After validating in an independent large cohorts and selecting miR-92a-3p as target miRNA to evaluate the effect of reference gene, we propose that combination of miR-191-5p and U6 could be used as reference genes for serum microRNAs qPCR data in colorectal adenocarcinoma, colorectal adenoma and healthy controls.

## Introduction

Colorectal cancer (CRC) is one of the most leading causes of cancer-related death with increased incidence and mortality in the past several decades. The progression to CRC is considered as a stepwise process with the accumulation of different genetic and epigenetic alterations resulting in a transformation from normal mucosa to precancerous lesion and finally to malignant tumor that is a well known normal mucosa-adenoma-adenocarcinoma (NM-A-AC) sequence [1]. Currently, several CRC screening studies pay more attention to the detection rate of precancerous adenoma which is associated with a high risk of progression to invasive lesion and represents the optimal target lesion for preventing CRC [2-4]. There is an urgent need to seek new biomarkers which should have high sensitivity and specificity for early-stage CRC and precancerous lesions.

MicroRNAs(miRNAs) are single-stranded RNA molecules, approximately 19-24 nucleotides in length, that regulate gene expression at the post-transcriptional level. Recently, serum miRNAs have been shown to be stable and reproducible which open a new opportunity of non-invasive test for the early diagnosis of cancer [5-7]. Quantitative real time polymerase chain reaction (qPCR) is the most frequently used approach for measurement of serum miRNAs due to its accuracy, sensitivity, specificity, reproducibility and robustness [8,9]. The accuracy of miRNAs expression analysis depends largely on a proper normalization. The use of reference genes as endogenous control is the most common method for normalizing qPCR data of miRNAs expression. The identification of suitable reference genes play a crucial role in miRNAs research because normalization to unreliable reference genes may lead to incorrect determination of miRNAs of interests [10,11]. The chosen of reference genes for detection of serum miRNAs is a major issue to be solved.

It has become clear that no single reference gene is constitutively expressed in all sample types, different kinds of disease and under all experimental designs which indicates that the expression stability of reference genes has to be verified before each experiment [12,13]. As far as aware, no systematic evaluation and validation of reference genes for normalizing qPCR analysis of serum miRNAs in colorectal adenocarcinoma has been published. In this study, we firstly made an effort to screen candidate reference genes using Miseq sequencing. Secondly, the reliability of candidate reference genes for normalization was evaluated by qPCR assays to select the most suitable reference genes. Then the selected reference genes were validated in an independent cohort study. To test for the effect of reference genes, we selected serum miR-92a-3p as target miRNA.

## Materials and Methods

### Ethics statement

Written informed consent was obtained from every participants for the use of the venous blood samples in this study. This project was approved by the Clinical Research Ethics Committee of Qilu Hospital of Shandong University.

### Study design and subjects

All the colorectal adenocarcinoma and adenoma patients were recruited from Department of General surgery and Gastroenterology, Qilu Hospital of Shandong University between 2010 and 2013. And the healthy controls were recruited from the Department of Physical Examination Center, Qilu Hospital of Shandong University.

We divided our study into three phases (Figure S1). The first phase was designed to screen candidate reference genes in serum of 3 pooling samples (30 patients with colorectal adenocarcinoma, 25 patients with colorectal adenoma and 30 healthy controls, respectively) using Miseq sequencing. Only the miRNAs which showed no differential expression among the 3 pooling samples were selected as candidate reference genes. The second phase was designed to evaluate the reliability of candidate reference genes for normalization by qPCR assays in an independent cohort of 125 samples (including 45 colorectal adenocarcinoma patients, 40 colorectal adenoma patients and 40 healthy controls) and to select the most suitable reference genes. Then the third phase was designed to validate the selected reference genes in another independent cohort of 128 colorectal adenocarcinoma patients, 60 colorectal adenoma patients and 60 healthy controls.

All the diagnosis of patients with colorectal adenocarcinoma and adenoma were confirmed by histopathology or histobiopsy. Tumors were staged according to the tumor-node-metastasis (TNM) staging system of Union for International Cancer Control (UICC). Healthy controls were recruited from a large pool of individuals seeking a routine health checkup who showed no evidence of disease and matched to the patients by age, sex (Table 1). All blood samples of patients were collected before any therapeutic procedures such as surgery, chemotherapy, and radiotherapy.

**Table 1:** Clinicopathological data for patients and healthy controls.

Characteristics		Screening phase	Selection phase	Validation phase
Healthy control	number	30	40	60
Age	mean±SD	54.37±16.24	56.38±15.40	56.30±16.82
Sex	Male(%)	18(60.0)	23(57.5)	37(61.7)
	Female(%)	12(40.0)	17(42.5)	23(38.3)
Colorectal adenoma	number	25	40	60
Age	mean±SD	54.60±17.36	57.30±14.09	57.81±14.42
Sex	Male(%)	16(64.0)	26(65.0)	35(58.3)
	Female(%)	9(36.0)	14(35.0)	25(41.7)
Location of adenoma	Colon(%)	18(72.0)	28(70.0)	46(76.7)
	Rectum(%)	7(28.0)	12(30.0)	14(23.3)
Colorectal adenocarcinoma	number	30	45	128
Age	mean±SD	56.56±19.67	57.0±17.21	55.62±16.43
Sex	Male(%)	19(63.3)	25(55.6)	73(57.0)
	Female(%)	11(36.7)	20(44.4)	55(43.0)
Location of tumor	Colon(%)	22(73.3)	32(71.1)	89(69.5)
	Rectum(%)	8(26.7)	13(28.9)	39(30.5)
Tumor size	≤3cm(%)	4(13.3)	9(20.0)	22(17.2)
	>3cm(%)	26(86.7)	36(80.0)	106(82.8)
Differentiation	Well(%)	4(13.3)	7(15.6%)	19(14.8)
	Moderate(%)	19(63.3)	25(55.5)	81(63.3)
	Poor(%)	5(16.7)	10(22.2)	28(21.9)
	N/A(%)	2(6.7)	3(6.7)	
TNM Stage	StageⅠ(%)	11(36.7)	16(35.5)	32(25.0)
	StageⅡ(%)	8(26.6)	12(26.6)	32(25.0)
	StageⅢ(%)	5(16.7)	7(15.6)	32(25.0)
	StageⅣ(%)	4(13.3)	7(15.6)	32(25.0)
	N/A(%)	2(6.7)	3(6.7)	

### Serum preparation

5ml venous blood was collected from each subject. The whole blood was separated into serum and cellular fractions by centrifugation at 4000 rpm for 10min, followed by 12000 rpm high-speed centrifugation for 15 min to completely remove cell debris. The entire process was finished within 2h after blood extraction and the supernatant serum was stored at -80°C until analysis.

### Miseq sequencing

For Miseq sequencing, equal volumes of serum from 30 patients with colorectal adenocarcinoma, 25 patients with colorectal adenoma and 30 healthy controls with similar age and sex distributions were pooled separately. Total RNA was extracted and purified using the miRNeasy Mini Kit (Qiagen, Valencia, CA) according to the manufacturer’s protocols. Briefly, a pair of adaptors was ligated to the 3’ and 5’ ends of miRNA, and miRNA molecules were amplified by qPCR to establish cDNA library. The quality of library was evaluated using KAPA qPCR kit by the two criteria: (a) the concentration of cDNA was more than 1nM, (b) showing no dimer contamination. Then the purified cDNA was used directly for sequencing analysis using Miseq sequencer (Illumina, San Diego, CA, USA) according to the manufacturer’s instructions. After a bioinformatics analysis, the remaining miRNAs were searched against the miRBase 17.0 to identify known mature miRNAs.

### Candidate reference genes

For Miseq sequencing, the final reads of each miRNA in each pooled sample were determined by normalization with the total reads of all called miRNAs in the sample. The miRNAs were selected as candidate reference genes that satisfied the following criteria: (a) having at least 50 copies in the three pooling samples, (b) showing no differential expression among three groups (*P*>0.05).

### qPCR analysis

qPCR was performed on ABI PRISM 7500 Sequence Detection System using the SYBR PrimeScript miRNA QPCR Kit (Takara Bio Inc). The reverse transcription (RT) reaction consisted of 10μl of 2× miRNA Reaction Buffer Mix, 2μl of miRNA Primescript RT Enzyme Mix, 2μl of 0.1% BSA and 3μl serum that mixed with 3μl serum buffer(2.5%Tween 20, 50 mmol/L Tris and 1mmol/L EDTA [14]). The reactions were incubated at 37°C for 60min, followed by 85°C for 5s and 4°C for 60min. The cDNA was centrifugated at 10000 rpm. for 10min. The PCR reaction consisted of 12.5μl of SYBR Premix Ex TaqⅡ, 0.5μl of DyeⅡ, 2μl of 5μM of forward primer, 1μl of 10μM of Uni-miR qPCR Primer, 7μl of ddH_2_O and 2.0μl of template cDNA into a total volume of 25μl. The reactions were incubated at 95°C for 30s, followed by 45 cycles of 95 °C for 5s and 57°C for 34s. To verify that the used primer pair produced only a single product, a dissociation protocol was added after thermocycling, determining dissociation of the PCR products from 65°C to 95°C. All reactions were performed in triplicate and the Cq value was determined using the fixed threshold setting. We used three criteria to identify candidate reference genes from the qPCR data: (a) the miRNA must be expressed in all samples; (b) mean Cq of the miRNA must be below 35; (c) there is no evidence for differential expression among the three groups.

### Data analysis

Statistical analysis was performed with SPSS 17.0 and Minitab 15 software and *P*<0.05 was considered statistically difference. One-way ANOVA test was used to compare the expression of candidate reference genes among colorectal adenocarcinoma, adenoma and healthy controls. Average values of triplicate Cq values were converted to relative quantities for GeNorm and Normfinder software to select the most stable reference genes. To calculate the expression of the target miR-92a-3p relative to suitable reference gene, 2^-△△Ct^ method was used. Mann-Whitney U test was used to compare the expression of miR-92a-3p among the three clinical groups.

## Results

### Selection of candidate reference genes by Miseq sequencing

Sequencing analysis was performed using Illumina’s Miseq sequencer and the clean readouts were compared with the miRBase 17.0 database. Of the 740 serum miRNAs that were scanned by sequencing, 348, 303 and 283 miRNAs were detectable (more than 10 copies) in colorectal adenocarcinoma patients, colorectal adenoma patients and healthy controls, respectively. For Miseq sequencing, the final reads of each miRNA in each pooled sample were determined by normalization with total reads of all called miRNAs. There were 17, 32 and 25 miRNAs having more than 50 copies and showing no differential expression among pooling group of colorectal adenocarcinoma and colorectal adenoma, colorectal adenocarcinoma and healthy controls, colorectal adenoma patients and healthy controls, respectively (*P*>0.05). According to the criteria described in “Material and Methods”, a list of 13 miRNAs was considered as candidate reference genes (Table 2).

**Table 2:** Details of candidate reference genes and target miRNA.

Name		Mature miRNA sequence	Copies		
			colorectal adenocarcinoma	colorectal adenoma	healthy control
Candidate reference gene	miR-103b	UCAUAGCCCUGUACAAUGCUGCU	10191	9121	10070
	miR-484	UCAGGCUCAGUCCCCUCCCGAU	3984	4222	3655
	miR-16-5p	UAGCAGCACGUAAAUAUUGGCG	3426	3228	3266
	miR-3615	UCUCUCGGCUCCUCGCGGCUC	2099	1915	2077
	miR-18a-3p	ACUGCCCUAAGUGCUCCUUCUGG	1557	1504	1494
	miR-197-3p	UUCACCACCUUCUCCACCCAGC	808	814	794
	miR-191-5p	CAACGGAAUCCCAAAAGCAGCUG	520	524	507
	miR-151a-3p	CUAGACUGAAGCUCCUUGAGG	157	167	160
	miR-26a-5p	UUCAAGUAAUCCAGGAUAGGCU	142	133	139
	miR-4446-3p	CAGGGCUGGCAGUGACAUGGGU	113	109	104
	miR-221-3p	AGCUACAUUGUCUGCUGGGUUUC	69	65	71
	miR-93-5p	CAAAGUGCUGUUCGUGCAGGUAG	52	58	55
	miR-3184-3	AAAGUCUCGCUCUCUGCCCCUCA	53	52	58
Target miRNA	miR-92a-3p	UAUUGCACUUGUCCCGGCCUGU	53088	19481	9440

### Confirmation of candidate reference genes by qPCR

**Figure 1:**
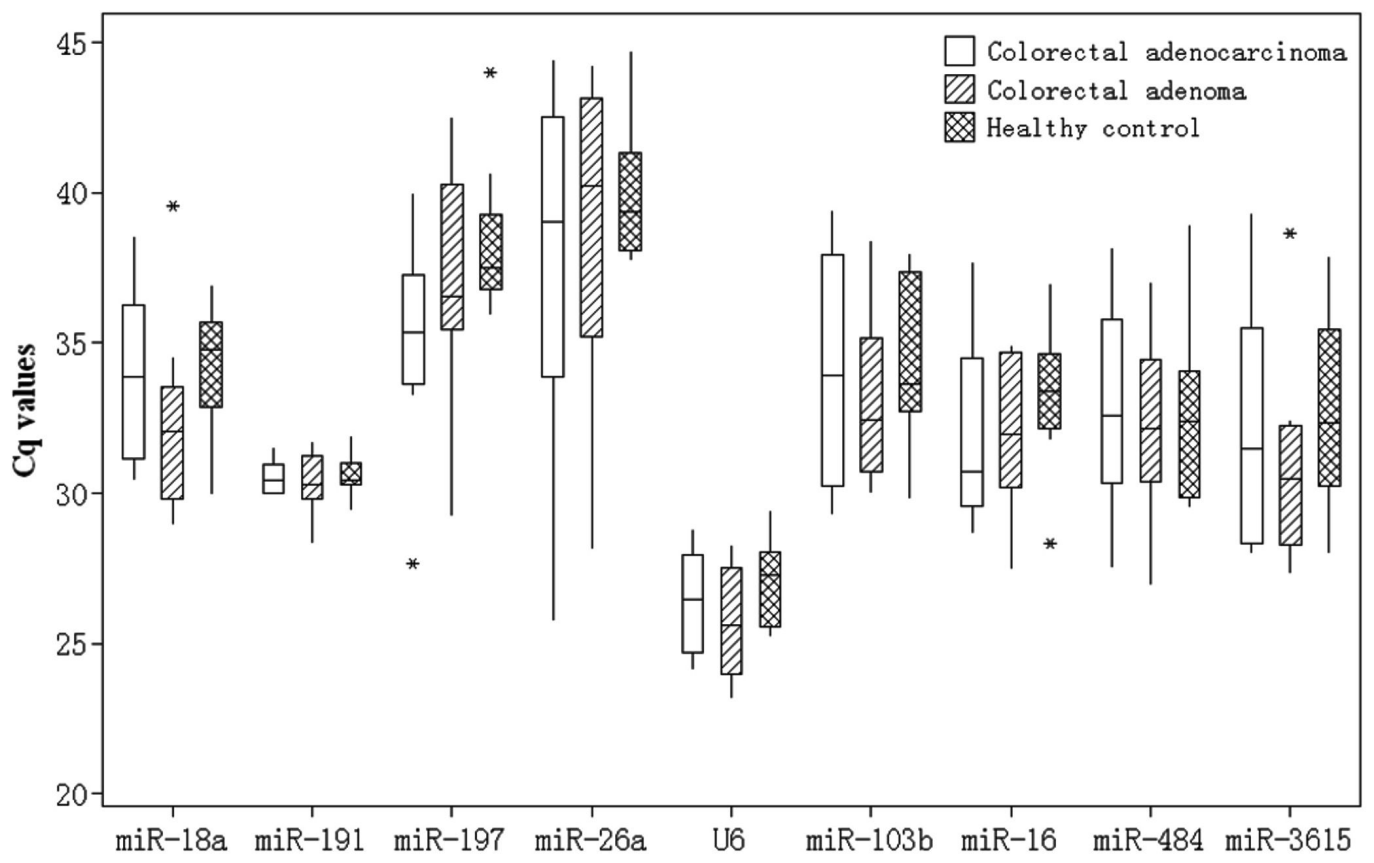
Cq values of candidate reference genes. Cq values of candidate reference genes in colorectal adenocarcinoma, colorectal adenoma and healthy control groups. No significant difference was found among three groups (*P*>0.05, ANOVA).

The above-mentioned 13 candidate reference genes and U6 were included in further confirmation phase because U6 was frequently used as reference gene in previous studies. According the three criteria described in the method, five miRNAs (miR-151a-3p, miR-4446-3p, miR-221-3p, miR-93-5p and miR-3184-3p )were not detected in all samples that were excluded from further analysis. Using the Cq values, there was no evidence for differential expression of any of the nine candidate reference genes among three groups (*P*>0.05) (Table 3 and Figure 1). The candidate reference genes displayed a wide expression range, with Cq values between 23.21 and 44.68 (Table 3). Expression of miR-197-3p and miR-26a-5p was relatively low, with median Cq more than 35 that were also excluded from further stability analysis. 

**Table 3:** Cq values of candidate reference genes.

Name	Cq Range	Cq Min	Cq Max	Mean Cq±SD			*P* value
				Colorectal adenocarcinoma	Colorectal adenoma	Healthy control	
miR-103b	10.05	29.34	39.39	33.10±2.78	34.04±3.83	34.29±2.72	0.306
miR-484	11.41	26.98	38.39	32.24±2.63	32.94±3.43	32.50±2.96	0.732
miR-16-5p	10.17	27.48	37.65	32.12±2.63	31.83±3.04	33.20±2.39	0.535
miR-3615	11.89	27.38	39.27	30.98±3.36	32.15±4.02	32.61±3.12	0.242
miR-18a-3p	10.54	29.01	39.55	32.33±3.20	33.82±2.89	34.18±2.15	0.5
miR-197-3p	16.36	27.64	44	37.17±3.89	35.02±3.45	38.25±2.52	0.135
miR-191-5p	3.49	28.37	31.86	30.34±1.02	30.40±5.78	30.59±0.68	0.793
miR-26a-5p	18.9	25.78	44.68	38.89±5.20	37.59±5.99	39.93±2.30	0.588
U6	6.15	23.21	29.36	25.55±1.84	26.34±1.72	27.01±1.40	0.199

### Expression stability analysis of selected reference genes by GeNorm and NormFinder

**Figure 2:**
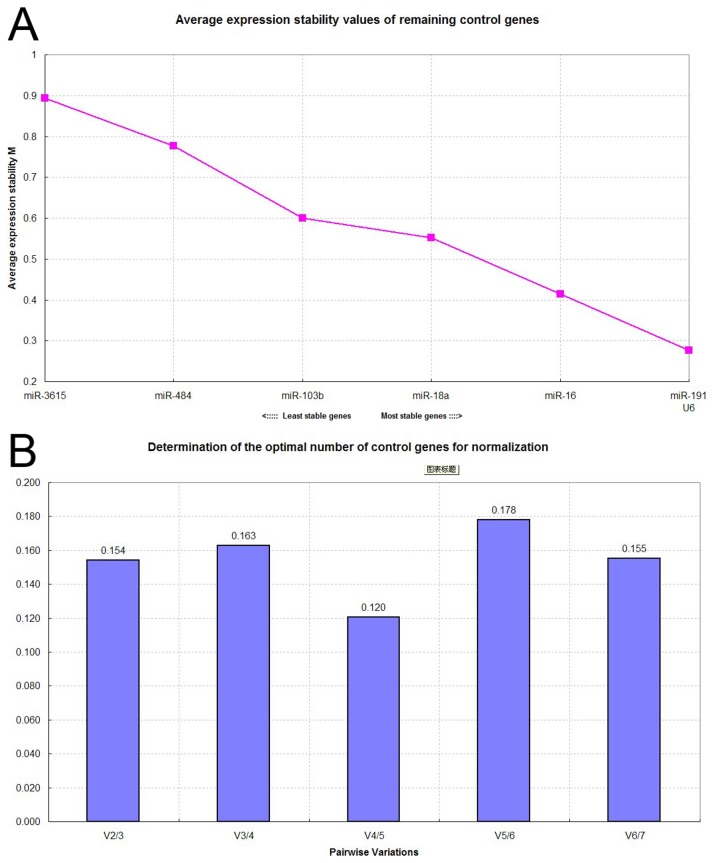
GeNorm analysis of candidate reference genes. (A) Ranking of candidate reference genes according to average expression stability. The least stable gene with the highest stability measure. M were excluded in a stepwise manner until two most stable genes remained: miR-191-5p and U6. (B) Determination of optimal number of reference genes for normalization. The geNorm output file indicated that optimal normalization of gene expression could be achieved using the top four most stable reference genes.

Variable stability of selected reference genes (miR-103b, miR-484, miR-16-5p, miR-3615, miR-18a-3p, miR-191-5p and U6) was evaluated using two algorithms: geNorm and NormFinder. The ranking of reference genes by the two programs is summarized in Table 4. Lower stability values characterize greater stability. GeNorm generates a gene stability value (M) and ranks candidate reference genes according to average expression stability accompanied by stepwise exclusion of the least stable gene until the two most stable genes remained (Figure 2). It also generates a normalization factor (NF) which is used to determine the optimal number of reference genes for normalization. This factor is calculated using the variable V as the pairwise variation (V_n_/V_n+1_) between two sequential NF_s_ (NF_n_ and NF_n+1_). The number of reference genes is considered optimal when the V value achieves the lowest. GeNorm recommended the use of four (miR-191-5p, miR-16-5p, U6 and miR-18a-3p) of the seven most stable genes for optimal normalization. NormFinder and geNorm both identified miR-191-5p as the most stably expressed reference genes and selected miR-191-5p and U6 as the most stable pair of reference gene.

**Table 4:** Ranking and best combination of candidate reference genes based on expression stability calculated by NormFinder and geNorm.

Rank	NormFinder		geNorm	
	gene	stablity	gene	Stablity(M)
1	miR-191-5p	0.494	miR-191-5p	0.677
2	miR-18a-3p	0.515	U6	0.715
3	U6	0.528	miR-16-5p	0.742
4	miR-16-5p	0.592	miR-18a-3p	0.854
5	miR-103b	0.605	miR-103b	0.925
6	miR-484	0.706	miR-484	1.156
7	miR-3615	0.792	miR-3615	1.187
Best combination	miR-191-5p/U6	0.363	miR-191-5p/U6	0.277

### Validation of suitable reference genes in a cohort samples

**Figure 3:**
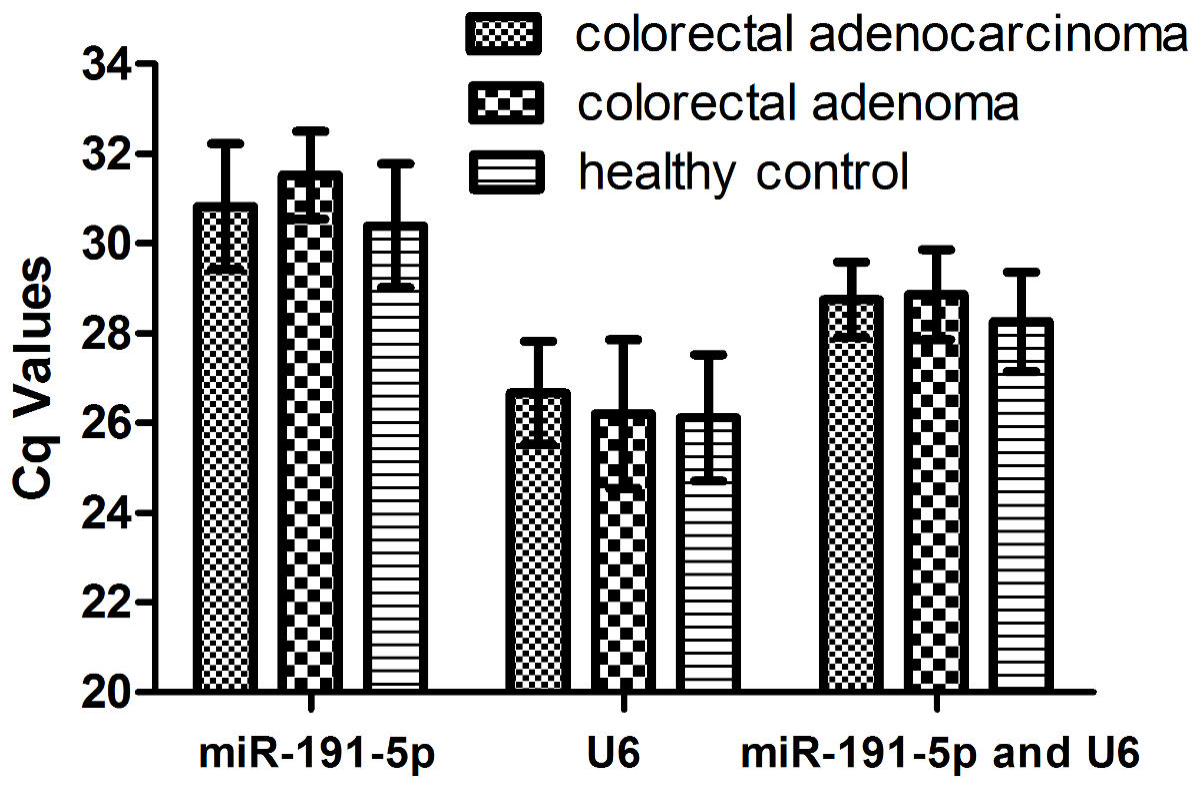
Cq values of miR-191-5p, U6, combination of miR-191-5p and U6 in three groups. No significant difference was found within 3 reference genes among the three groups (*P*>0.05, ANOVA).

**Figure 4:**
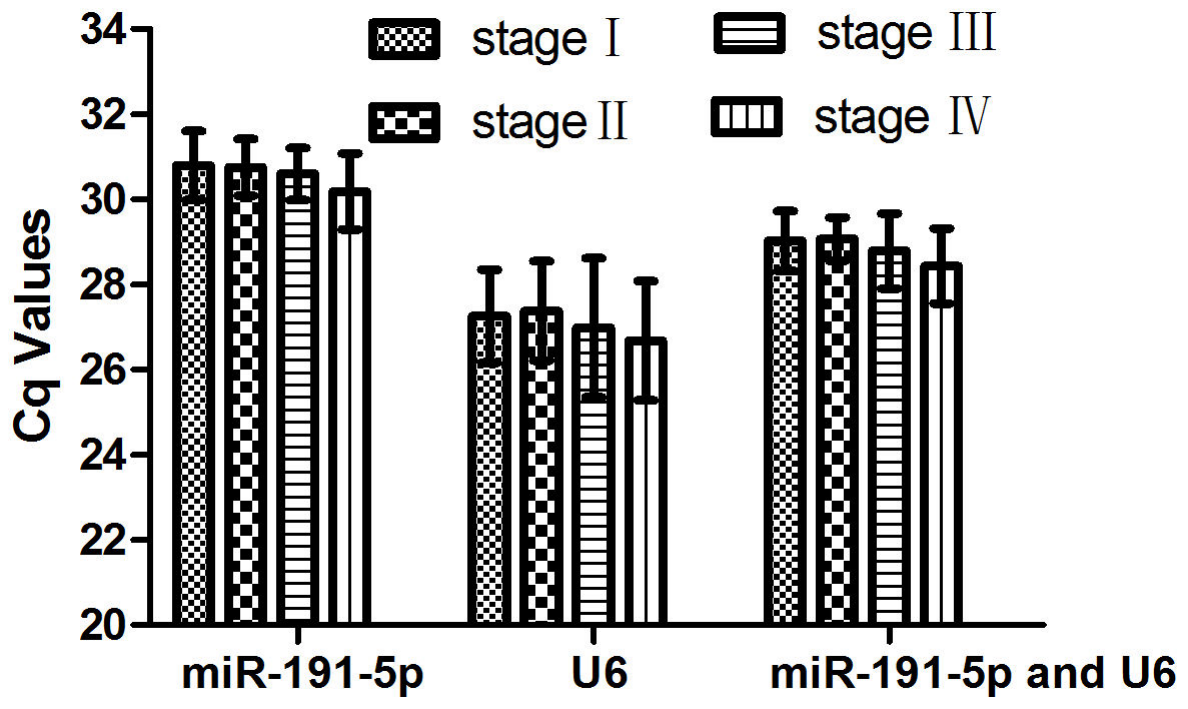
Cq values of miR-191-5p, U6, combination of miR-191-5p and U6 in different stages of colorectal adenocarcinoma group. No significant difference was found within 3 reference genes among the four stages (*P*>0.05, ANOVA).

In order to further validate the stability of miR-191-5p, U6 and combination of these two genes as reference genes, we applied an independent cohort study comprised of 128 colorectal adenocarcinoma patients, 60 colorectal adenoma patients and 60 healthy controls. The expression level of miR-191-5p, U6 and combination of miR-191-5p and U6 (mean) in three groups and four stages of colorectal adenocarcinoma group were shown in Figure 3 and Figure 4. Using the Cq values of each validate reference gene, there was no evidence for differential expression among the three groups and four stages of colorectal adenocarcinoma. 

### Effect of suitable reference genes on relative quantity of serum miR-92a-3p

**Figure 5:**
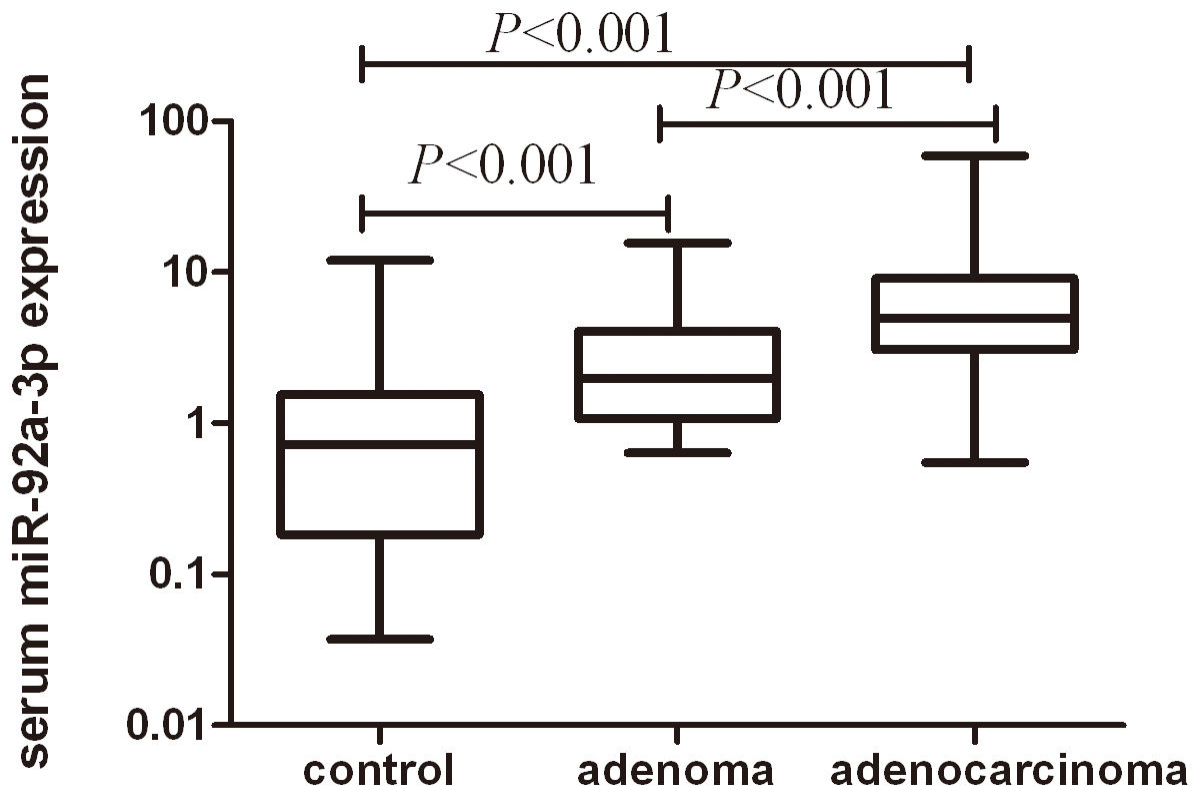
Serum miR-92a-3p expression in three groups. Our results indicated that serum miR-92a-3p was significantly higher in colorectal adenocarcinoma patients than colorectal adenoma patients and healthy controls (*P*<0.001). The difference in miR-92a levels remained significant between colorectal adenoma patients and healthy controls (*P*<0.001).

**Figure 6:**
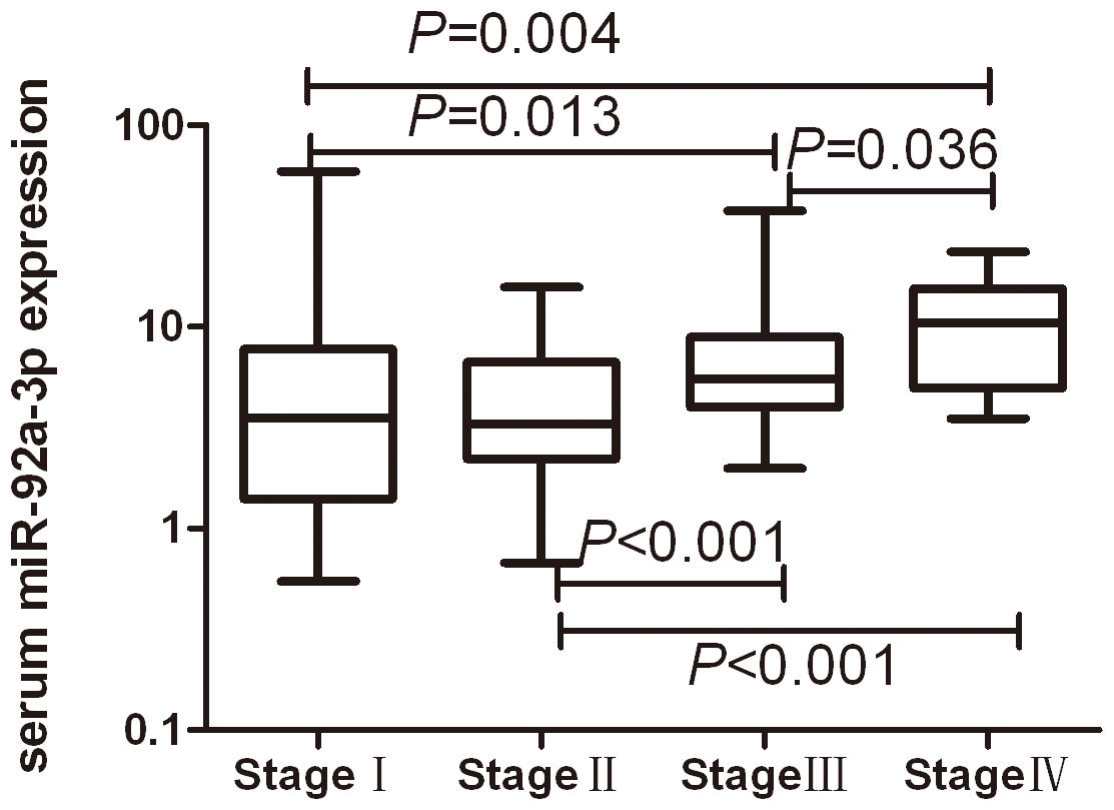
Serum miR-92a-3p expression in in different stages of colorectal adenocarcinoma group. The expression of miR-92a-3p increased with the progress of TNM staging. There was significant difference of serum miR-92a-3p between StageⅠ and StageⅢ/Ⅳ, between StageⅡ and StageⅢ/Ⅳ, between StageⅢ and StageⅣ. The difference of serum miR-92a-3p between StageⅠ and StageⅡ was not found (*P*=0.66).

We evaluated miR-92a-3p expression using combination of miR-191-5p and U6 (mean) as reference gene in the serum of colorectal adenocarcinoma, colorectal adenoma and healthy controls. Our results indicated that serum miR-92a-3p was significantly higher in colorectal adenocarcinoma patients than colorectal adenoma patients and healthy controls (*P*<0.001). The difference in miR-92a-3p level remained significant between colorectal adenoma patients and healthy controls(*P*<0.001) (Figure 5). The expression of miR-92a-3p increased with the progress of TNM staging (Figure 6). There was significant difference of serum miR-92a-3p between StageⅠ and StageⅢ/Ⅳ, between StageⅡ and StageⅢ/Ⅳ, between StageⅢ and Stage Ⅳ. The difference of serum miR-92a-3p between StageⅠ and Stage Ⅱ was not found (*P*=0.66).

## Discussion

Serum microRNAs as a class of novel promising non-invasive biomarkers for cancer diagnosis have been demonstrated by many studies [15-17]. They are stable, reproducible, easy to access and potentially cancer specific. General technologies for the analysis of miRNAs expression include chip-based microarrays, next generation sequencing and qPCR. High-throughput methods such as microarray and sequencing enable simultaneous detection of hundreds of miRNAs in a single sample. However, the meaningful interpretation of such large datesets has to be validated by method of qPCR [18,19]. The reliable results of qPCR demand appropriate normalization to minimize variation that can mask or exaggerate meaningful changes. The utility of reference genes as endogenous control is the most common method for normalizing qPCR data of miRNAs expression [20]. 

In a previous study, miRNA-484 and miRNA-191 were identified as the most stable pair of endogenous control for serum miRNAs detection in breast cancer [21]. Another report recommended the combination of miR-16 and miR-93 as suitable reference genes for serum miRNAs analysis in gastric cancer patients and healthy controls [22]. Kah [11] demonstrated in colorectal tissue that despite a relatively large sample size, when inappropriate reference genes were used for normalization, a true biological difference in expression between tumor and normal was not detected. These results highlight the importance of selecting appropriate reference genes in miRNAs expression study. To our knowledge, this is the first report detailing identification and validation of suitable reference genes for normalization of serum miRNAs qPCR data for colorectal adenocarcinoma. 

We firstly profiled pooled serum of colorectal adenocarcinoma, colorectal adenoma and healthy controls and selected a list of 13 miRNAs as candidate reference genes. U6 belongs to a class of small non-coding RNA and cannot be sequenced by Miseq technology in our designed program for miRNAs sequencing. Therefore, U6 didn’t appear in the screening phase. It was previously used as endogenous control for almost all the tissue miRNAs analysis. However, using U6 to normalize serum miRNAs levels is controversial because it was reported not suitable for serum miRNAs normalization in several studies while chosen as stable reference gene in other studies [23,24]. It was demonstrated that U6 was stable in CRC patients and healthy controls in our preliminary test. Thus, U6 and above-mentioned 13 miRNAs were included in further confirmation by qPCR. According to the three criteria that we have demonstrated in our results, 7 miRNAs (miR-151a-3p, miR-4446-3p,miR-221-3p, miR-93-5p and miR-3184-3p, miR-197-3p and miR-26a-5p) were excluded from further stability analysis. Then variable stability of the other 6 miRNAs (miR-103b, miR-484, miR-16-5p, miR-3615, miR-18a-3p and miR-191-5p) and U6 were evaluated using two algorithms: geNorm and NormFinder. The geNorm is a pair wise comparison model while NormFinder is an ANOVA-based model. They are two distinct statistical models that have been widely used for selecting optimal reference genes in normalizing gene expression. One important difference between the geNorm and NormFinder model is that NormFinder allows designation of sample groups and determines both intragroup and intergroup variation [25]. GeNorm recommended the use of four most stable genes (miR-191-5p, miR-16-5p, U6 and miR-18a-3p) for optimal normalization. Both NormFinder and geNorm both identified miR-191-5p as the most stably expressed reference gene and selected miR-191-5p and U6 as the most stable pair of reference gene.

In order to validate the selected reference genes, an independent large cohort was applied to evaluate the stability of reference genes. Our result demonstrated that there was no evidence for differential expression of miR-191-5p, U6 and combination of miR-191-5p and U6 (mean) among the three groups. Furthermore, serum miRNAs levels can vary highly depending on the tumor stages. Thus, we also evaluated the stability of reference gene in different stages of colorectal adenocarcinoma. The results indicated that there was no evidence for differential expression of miR-191-5p, U6 and combination of miR-191-5p and U6 (mean) among the four stages.

Several studies evaluated the effect of selected reference genes as endogenous control by analyzing one or certain target miRNAs which has been shown to be upregulated or downregulated in previous reports [22,26]. It has been reported that miR-92a was up-regulated in plasma of patients with colorectal cancer and could be used as a marker for colorectal cancer screening in previous studies [2,27]. Furthermore, in our sequencing result, miR-92a-3p was higher in colorectal adenocarcinoma group than in colorectal adenoma group (*P*=2.964e-323) and in healthy controls (*P*=1.482e-323). There was also significant difference of miR-92a-3p between colorectal adenoma group and healthy controls (*P*=2.47e-323). Therefore, we selected miR-92a as the target miRNA to test whether it has the similar discrimination effect among healthy, colorectal adenoma, and colorectal adenocarcinoma patients when using combination of miR-191-5p and U6 as reference genes. Our results were not completely consistent with the previous studies. Using combination of miR-191-5p and U6 as reference gene, miR-92a-3p could not only efficiently discriminate healthy controls, colorectal adenoma and colorectal adenocarcinoma patients but also find the minor difference between different stages of colorectal adenocarcinoma which were not found normalized to miR-16 and RNU6B in previous study [2,27].

It has been reported that using of more than one reference genes increases the accuracy of quantization comparing to a single reference gene. Thus, we propose that combination of miR-191-5p and U6 could be used as reference genes for serum microRNAs qPCR study in patients with colorectal adenocarcinoma, adenoma and healthy controls.

## Supporting Information

Figure S1 **Workflow chart of the study design.**
(TIF)
