# Supplementary figures and images for: Identification and Validation of Reference Genes for qPCR Detection of Serum microRNAs in Colorectal Adenocarcinoma Patients
Source: PLoS One. 2013 Dec 11;8(12):e83025. doi: 10.1371/journal.pone.0083025 (PMC3859607; doi:10.1371/journal.pone.0083025)

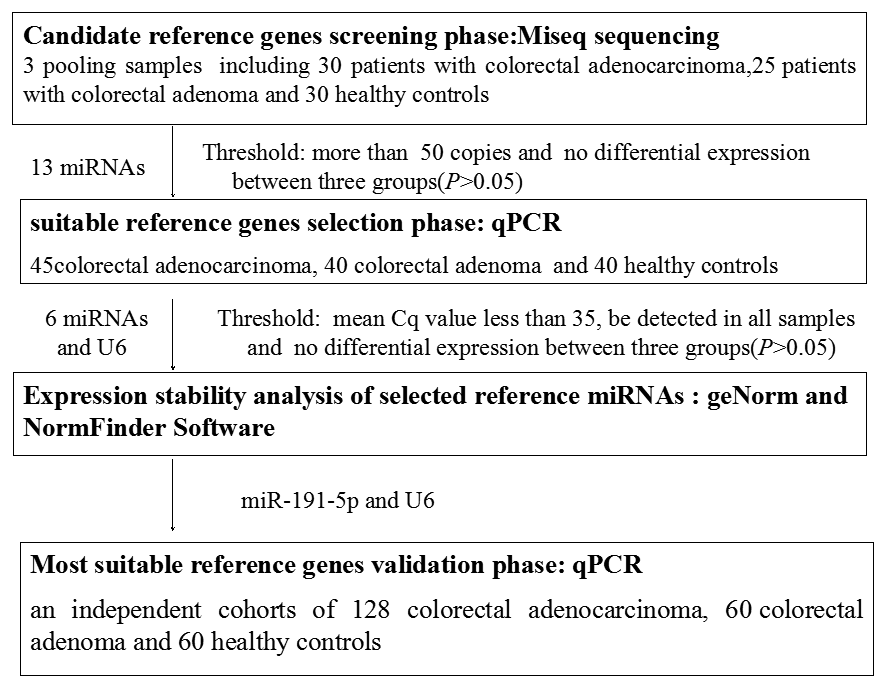

Supplement: Figure S1 — Workflow chart of the study design. (TIF) [file pone.0083025.s001.tif]
